# Real-world safety profile of mosunetuzumab: a pharmacovigilance study based on the food and drug administration adverse event reporting system

**DOI:** 10.3389/fphar.2026.1809879

**Published:** 2026-05-26

**Authors:** Jiahui Liu, Yingxi Zhang, Ting Yang, Ling Zhang, Yanhong Luo, Junyan Zhang, Ruijuan Zhang

**Affiliations:** 1 Department of Health Statistics, School of Public Health, Shanxi Medical University, Taiyuan, China; 2 Department of Hematology, Third Hospital of Shanxi Medical University, Shanxi Bethune Hospital, Shanxi Academy of Medical Sciences, Tongji Shanxi Hospital, Taiyuan, China; 3 Department of Clinical Epidemiology and Evidence-Based Medicine, Shanxi Bethune Hospital, Academy of Medical Sciences, Tongji Shanxi Hospital, Taiyuan, China; 4 Department of Hematology, The First Hospital of Shanxi Medical University, Taiyuan, China; 5 Research Center for Hemostatic Disorders and Hematologic Malignancies, Shanxi Medical University, Taiyuan, China

**Keywords:** adverse events, disproportionality analysis, FAERS, mosunetuzumab, pharmacovigilance

## Abstract

**Background:**

Mosunetuzumab is a CD20×CD3 bispecific antibody approved for adult relapsed or refractory follicular lymphoma. Post-marketing evidence on its safety profile remains scarce. This pharmacovigilance study used the Food and Drug Administration Adverse Event Reporting System (FAERS)to characterize real-world adverse event (AE) signals associated with mosunetuzumab.

**Methods:**

We performed disproportionality analysis using reports from the FAERS database spanning from the first quarter of 2004 to the fourth quarter of 2025. The primary analysis set encompassed reports wherein mosunetuzumab was coded as the Primary Suspect (PS). Disproportionality analyses were performed using four algorithms: reporting odds ratio (ROR), proportional reporting ratio (PRR), Bayesian confidence propagation neural network (BCPNN), and multi-item gamma poisson shrinker (MGPS). After excluding implausible records, the time to onset (TTO)for AEs was summarized using the median and interquartile range.

**Results:**

Overall, 1154 FAERS reports mentioning mosunetuzumab were included in the primary analysis. 3 positive signals were identified at the System Organ Class (SOC)level. Cytokine release syndrome (CRS), neutropenia, tumour flare and infection-related events were frequently reported signals at the Preferred Term (PT)level. Additional disproportionate reporting signals were noted for cardiac events and eye disorders, including atrial fibrillation, uveitis. Of 310 reports with valid dates, most AEs occurred within 30 days following treatment initiation, whereas a nontrivial proportion was reported at ≥180 days.

**Conclusion:**

This study offers real-world evidence for guiding the clinical use of mosunetuzumab. It highlights the necessity for clinicians to monitor for AEs during treatment by recognizing potential adverse reaction signals beyond those documented in the current prescribing information.

## 1 Introduction

Follicular lymphoma (FL), a prevalent form of B-cell non-Hodgkin lymphoma, originates from germinal center B cells and mainly involves the lymph nodes, bone marrow, and spleen. Epidemiologically, FL more frequently occurs among individuals from Western countries, with a median age at diagnosis of approximately 65 years and a slight male predominance (Mozas et al., 2021; Laurent et al., 2023; Cerhan, 2020). The development of targeted therapies, including rituximab, has significantly enhanced survival outcomes in patients with FL, increasing the 10-year overall survival (OS) rate to approximately 80% (Jacobsen, 2022). Nevertheless, the management of relapsed or refractory FL (R/R FL) remains a crucial clinical challenge.

Mosunetuzumab, as the first CD20 × CD3 bispecific antibody approved globally by the U.S., activates T cell-mediated tumour killing by bridging T cells with malignant B cells, providing a novel therapeutic option for patients with R/R FL (Bender et al., 2024; Kang, 2022). However, certain adverse reaction risks, particularly immune-related adverse events (AEs) such as cytokine release syndrome (CRS) accompany its clinical use (Chennapragada and Ramadas, 2024; Matasar et al., 2024).

Currently, existing real-world evidence remains inconclusive, and comprehensive systematic analyses based on large-scale pharmacovigilance databases remain scarce. The U.S. Food and Drug Administration Adverse Event Reporting System (FAERS) provides a valuable data source for relevant investigations. Therefore, this study conducted disproportionality analysis-based signal detection and hierarchical interpretation of AEs associated with mosunetuzumab utilizing FAERS data from the first quarter of 2004 to the fourth quarter of 2025. This study aimed to characterize its real-world safety profile and provide evidence for guiding clinical risk monitoring and rational drug use.

## 2 Methods

### 2.1 Data source and case selection

**FIGURE 1:**
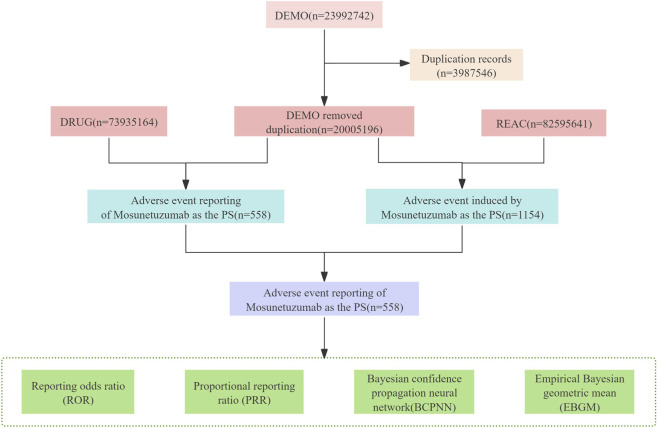
Flowchart of FAERS data extraction, deduplication, and selection of mosunetuzumab from 2004Q1 to 2025Q4.

This study constituted a retrospective analysis for AE signal detection utilizing a pharmacovigilance database. To identify records, individual case safety reports from the first quarter of 2004 to the fourth quarter of 2025 were extracted from the FAERS using its brand and generic names. The search terms included “MOSUNETUZUMAB,” and “LUNSUMIO.” Mosunetuzumab was first approved by the FDA in December 2022 (6); however, pre-approval reports (with FDA receipt dates as early as 2019) are also present in the database, primarily originating from clinical trials and early access programs. The FAERS database comprises the following seven tables: demographic (DEMO), drug (DRUG), report sources (RPSR), therapy (THER), indication (INDI), reaction (REAC), and outcome (OUTC). Reported drugs in FAERS are classified into the following four groups: Primary Suspect (PS), Secondary Suspect (SS), Concomitant (C), and Interacting (I). The primary analysis set was restricted to reports where mosunetuzumab was designated as the PS. Reports where mosunetuzumab was listed as Secondary Suspect (SS), Concomitant (C), or Interacting (I) were excluded from the primary analysis. AEs were coded using the Medical Dictionary for Regulatory Activities (MedDRA) and analyzed at both the System Organ Class (SOC) and Preferred Term (PT) levels. Data cleaning adhered to the FDA-recommended deduplication strategy. From the DEMO table, we selected the PRIMARYID, CASEID, and FDA-DT fields, extracting the maximum FDA-DT value according to FDA guidelines to ensure that the most recent report for each CASEID was retained. We retained the highest PRIMARYID when the FDA-DT and CASEID were identical. The design flowchart is illustrated in Figure 1. Furthermore, terms not directly indicative of adverse drug reactions (e.g., product issues and medication errors) were excluded before the final analysis and presentation of key results.

### 2.2 Statistical analysis

Disproportionality analysis, which frequently includes two components (frequentist and Bayesian statistics), was employed for signal detection. Frequentist statistics comprises the reporting odds ratio (ROR) and proportional reporting ratio (PRR), whereas Bayesian statistics encompasses the multi-item gamma poisson shrinker (MGPS) and Bayesian confidence propagation neural network (BCPNN). In this study, AEs that met the positive thresholds across all four methods were categorized as positive signals (Evans et al., 2001; Hauben et al., 2005; Weinstein et al., 2009; Kuai et al., 2026). This unified positive signal determination standard is applicable to both the SOC level and the PT level analysis. Combining four algorithms reduced statistical bias generated by employing only one method. Signal strength and frequency were summarized at both the SOC and PT levels. Time to onset was defined as the time interval between the AE onset date recorded in the DEMO file and the start date of mosunetuzumab therapy recorded in the THER file. Records with negative or implausible time-to-onset values were excluded. Time to onset was summarized using median and interquartile range and further classified into prespecified windows (≤30, 31–90, 91–180, and >180 days). The 2 × 2 contingency table utilized in the disproportionality analysis is shown in Table 1. The algorithms and positive threshold criteria for the four methods are presented in Table 2. Of note, disproportionality analysis indicates a statistical association between the drug and the event, which does not establish a causal relationship. This study was exempt from Institutional Review Board approval and the requirement for informed consent as it analyzed fully anonymized data from the publicly available FAERS database and did not involve any direct intervention with human participants or access to identifiable private information.

**TABLE 1:** Two-by-two contingency table for disproportionality analyses.

Drug	Target AEs	Other AEs	Total
Target drug	a	b	a+b
Other drugs	c	d	c+d
Total	a+c	b+d	a+b + c+d

Abbreviation: AEs, adverse events; a, number of reports containing both the target drug and target adverse drug reaction; b, number of reports containing other adverse drug reaction of the target drug; c, number of reports containing the target adverse drug reaction of other drugs; d, number of reports containing other drugs and other adverse drug reactions.

**TABLE 2:** Four algorithms used for signal detection.

Algorithms	Equation	Criteria
ROR	ROR = ad/bc	Lower limit of 95% CI > 1,N ≥ 3
	${95\%CI=e}^{\ln\left(ROR\right)\pm 1.96\sqrt{{\left(1/a+1/b+1/c+1/d\right)}^{0.5}}}$	
PRR	PRR = a (c + d)/c/(a+b)	PRR≥2,χ^2^ ≥ 4,N ≥ 3
	χ^2^ = [(ad-bc)^2^](a+b + c + d)/[(a+b) (c + d) (a+c) (b + d)]	
MGPS	EBGM = a (a+b + c + d)/(a+c)/(a+b)	EBGM05 > 2
	95% CI = $e^{\ln\left(EBGM\right)\pm 1.96{\left(1/a+1/b+1/c+1/d\right)}^{0.5}}$	
BCPNN	IC = $\log_{2}a\left(a+b+c+d\right)\left(a+c\right)\left(a+b\right)$	IC025 > 0
	95% CI = E (IC)±2 V(IC)^0.5^	

Abbreviation: a, number of reports containing both the target drug and target adverse drug reaction; b, number of reports containing other adverse drug reaction of the target drug; c, number of reports containing the target adverse drug reaction of other drugs; d, number of reports containing other drugs and other adverse drug reactions. 95% CI, 95% confidence interval; N:the number of reports; χ^2^, chi-squared; IC, information component; IC025, the lower limit of 95% CI of the IC; E (IC), the IC expectations; V(IC), the variance of IC; EBGM, empirical Bayesian geometric mean; EBGM05, the lower limit of 95% CI of EBGM.

## 3 Results

### 3.1 Baseline demographic and clinical characteristics

The FAERS database contained 23,992,742 reports spanning from the first quarter of 2004 to the fourth quarter of 2025. A total of 1,154 adverse event reports with mosunetuzumab as the primary suspect drug were retrieved from the FAERS database. After deduplication by the unique case identifier primaryid, 558 unique patients were identified. These 558 patients formed the study population for the statistical analysis (Table 3). Among reports with available sex information, males accounted for 266 cases (47.7%) and females for 210 cases (37.6%), while sex was missing in 82 reports (14.7%). Regarding age distribution, patients aged 65–85 years constituted the largest group (232, 41.6%), followed by those aged 18–64.9 years (170, 30.5%). Among the reported serious outcomes, hospitalization was the most frequently reported (272, 48.7%), followed by death (75, 13.4%), life-threatening events (17, 3.0%), and disability (1, 0.2%). Most reports were submitted by physicians (431, 77.2%) and pharmacists (44, 7.9%). Geographically, the United States (260, 46.6%) and Spain (28,5.0%) contributed the highest proportion of reports. An analysis of reporting trends over time revealed a progressive increase in AE reports following the drug’s market approval, peaking in 2023 (322, 57.7%).

**TABLE 3:** Demographic and reporting characteristics of mosunetuzumab-associated FAERS reports from 2004Q1 to 2025Q4.

Characteristics	Number	Proportion (%)
Number of reporters	558	​
Genders		
Female	210	37.6%
Male	266	47.7%
Missing	82	14.7%
Weight		
<50 kg	17	3.0%
50–100 kg	277	49.6%
>100 kg	30	5.4%
Missing	234	41.9%
**Age**	​	​
18–64.9	170	30.5%
65–85	232	41.6%
>85	10	1.8%
Missing	146	26.2%
Outcome		
Death	75	13.4%
Life-threatening	17	3.0%
Disability	1	0.2%
Hospitalization	272	48.7%
Other	193	34.6%
Reporter		
Physician	431	77.2%
Health professional	40	7.2%
Pharmacist	44	7.9%
Consumer	38	6.8%
Missing	5	0.9%
Reported countries		
United States	260	46.6%
Spain	28	5.0%
Japan	26	4.7%
France	23	4.1%
Brazil	20	3.6%
China	17	3.0%
Canada	14	2.5%
South Korea	12	2.2%
Other	158	28.4%
Report year		
2020	12	2.2%
2021	24	4.3%
2022	26	4.7%
2023	322	57.7%
2024	82	14.7%
2025	92	16.5%

### 3.2 Signal detection at the SOC level

**FIGURE 2:**
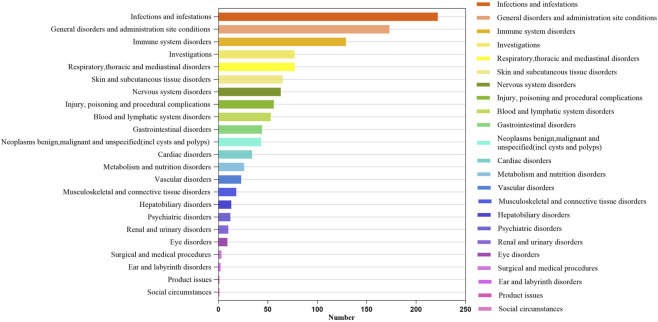
Distribution of mosunetuzumab-associated adverse event reports by SOC in FAERS.

The proportions of reported SOC among mosunetuzumab-related AEs are depicted in Figure 2. The signal detection results at the SOC level are summarized in Table 4. A total of 23 SOCs were reported. However, only three SOCs met the positive criteria across all four disproportionality methods (ROR, PRR, MGPS, and BCPNN): “Infections and infestations” (n = 222, ROR = 4.3), “Immune system disorders” (n = 129, ROR = 10.8), and “Blood and lymphatic system disorders” (n = 53, ROR = 2.86).

**TABLE 4:** SOC-level disproportionality results for mosunetuzumab in FAERS.

System organ class (SOC)	Reported Case	ROR (95% CI)	PRR (χ^2^)	EBGM (EBGM05)	IC (IC025)
Infections and infestations	222	4.3 (3.71–4.97)	3.66 (453.48)	3.66 (3.16)	1.87 (1.64)
General disorders and administration site conditions	173	0.83 (0.7–0.97)	0.85 (5.3)	0.85 (0.73)	−0.23 (−0.46)
Immune system disorders	129	10.8 (9–12.97)	9.71 (1019.1)	9.71 (8.08)	3.28 (2.92)
Investigations	77	1.11 (0.88–1.4)	1.1 (0.81)	1.1 (0.88)	0.14 (−0.2)
Respiratory, thoracic and mediastinal disorders	77	1.45 (1.15–1.82)	1.42 (9.92)	1.42 (1.12)	0.5 (0.16)
Skin and subcutaneous tissue disorders	65	1.01 (0.79–1.3)	1.01 (0.01)	1.01 (0.79)	0.02 (−0.35)
Nervous system disorders	63	0.63 (0.49–0.81)	0.65 (13.02)	0.65 (0.5)	−0.62 (−0.99)
Injury, poisoning and procedural complications	56	0.43 (0.33–0.56)	0.46 (40.47)	0.46 (0.35)	−1.13 (−1.51)
Blood and lymphatic system disorders	53	2.86 (2.17–3.77)	2.77 (61.11)	2.77 (2.11)	1.47 (1.02)
Gastrointestinal disorders	44	0.42 (0.31–0.57)	0.45 (33.28)	0.45 (0.33)	−1.17 (−1.59)
Neoplasms benign, malignant and unspecified (incl cysts and polyps)	43	1.59 (1.17–2.16)	1.57 (9.11)	1.57 (1.16)	0.65 (0.19)
Cardiac disorders	34	1.17 (0.83–1.65)	1.17 (0.83)	1.17 (0.83)	0.22 (−0.28)
Metabolism and nutrition disorders	26	1.04 (0.71–1.53)	1.04 (0.04)	1.04 (0.7)	0.06 (−0.51)
Vascular disorders	23	0.95 (0.63–1.43)	0.95 (0.06)	0.95 (0.63)	−0.07 (−0.67)
Musculoskeletal and connective tissue disorders	18	0.29 (0.18–0.46)	0.3 (31.16)	0.3 (0.19)	−1.74 (−2.35)
Hepatobiliary disorders	13	1.26 (0.73–2.18)	1.26 (0.7)	1.26 (0.73)	0.33 (−0.47)
Psychiatric disorders	12	0.18 (0.1–0.32)	0.19 (43.68)	0.19 (0.11)	−2.39 (−3.11)
Renal and urinary disorders	10	0.44 (0.24–0.82)	0.45 (7.04)	0.45 (0.24)	−1.17 (−1.97)
Eye disorders	9	0.39 (0.2–0.74)	0.39 (8.73)	0.39 (0.2)	−1.36 (−2.18)
Surgical and medical procedures	3	0.19 (0.06–0.59)	0.19 (10.37)	0.19 (0.06)	−2.38 (−3.5)
Ear and labyrinth disorders	2	0.39 (0.1–1.57)	0.39 (1.88)	0.39 (0.1)	−1.35 (−2.69)
Product issues	1	0.05 (0.01–0.36)	0.05 (17.51)	0.05 (0.01)	−4.26 (−5.38)
Social circumstances	1	0.19 (0.03–1.32)	0.19 (3.55)	0.19 (0.03)	−2.42 (−3.71)

95% CI, 95% confidence interval; χ^2^, chi-squared; IC, information component; IC025, the lower limit of 95% CI, of the IC; EBGM, empirical Bayesian geometric mean; EBGM05, the lower limit of 95% CI, of EBGM.

### 3.3 Signal detection at the PT level

**FIGURE 3:**
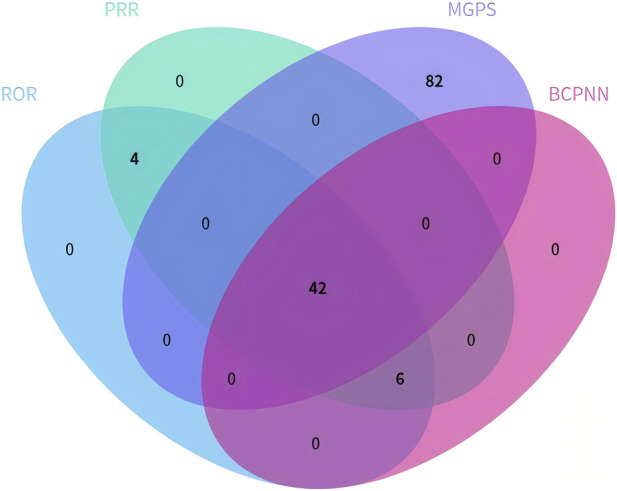
Overlap of PT level safety signals identified by four disproportionality methods for mosunetuzumab in FAERS.

At the PT level, 42 positive signals were identified using the four methods simultaneously (Figure 3). We excluded adverse events potentially unrelated to mosunetuzumab. Ultimately, 34 positive signals were identified, their signal strengths are summarized in Table 5. These high-frequency AEs were categorized with a clear classification of signal category (known risk or novel potential signal) for systematic interpretation as follows.

**TABLE 5:** PT level disproportionality signals for mosunetuzumab in FAERS.

Preferred terms (PTs)	Reported Case	ROR (95% CI)	PRR (χ^2^)	EBGM (EBGM05)	IC (IC025)
Cytokine release syndrome	118	484.63 (400.39–586.6)	435.18 (50,818.6)	432.56 (357.37)	8.76 (6.27)
Pyrexia	36	5.78 (4.15–8.06)	5.63 (137.87)	5.63 (4.04)	2.49 (1.84)
COVID-19 pneumonia	33	202.94 (143.49–287.03)	197.17 (6423.98)	196.63 (139.03)	7.62 (4.36)
COVID-19	29	11.78 (8.15–17.03)	11.51 (278.88)	11.51 (7.96)	3.52 (2.56)
Pneumonia	25	3.9 (2.62–5.79)	3.84 (52.7)	3.84 (2.58)	1.94 (1.22)
Neutropenia	21	8.8 (5.71–13.55)	8.66 (142.48)	8.65 (5.62)	3.11 (2.06)
Pneumonitis	16	32.73 (19.98–53.61)	32.29 (485.09)	32.27 (19.7)	5.01 (2.8)
Febrile neutropenia	13	11.04 (6.39–19.06)	10.92 (117.28)	10.92 (6.32)	3.45 (1.9)
Atrial fibrillation	13	7.29 (4.22–12.6)	7.22 (69.8)	7.22 (4.18)	2.85 (1.55)
Tumour flare	12	1019.84 (575.18–1808.27)	1009.25 (11,919.02)	995.23 (561.3)	9.96 (2.87)
Alanine aminotransferase increased	12	11.15 (6.31–19.69)	11.05 (109.72)	11.04 (6.25)	3.47 (1.83)
Sepsis	11	5.29 (2.92–9.58)	5.25 (37.88)	5.25 (2.9)	2.39 (1.12)
Injection site reaction	11	8.57 (4.73–15.52)	8.5 (72.83)	8.5 (4.69)	3.09 (1.55)
Aspartate aminotransferase increased	11	11.96 (6.61–21.66)	11.86 (109.42)	11.86 (6.55)	3.57 (1.8)
Infusion related reaction	11	9.86 (5.45–17.86)	9.78 (86.76)	9.78 (5.4)	3.29 (1.66)
Chills	10	4.65 (2.5–8.67)	4.62 (28.44)	4.62 (2.48)	2.21 (0.92)
Respiratory failure	8	5.93 (2.96–11.88)	5.89 (32.54)	5.89 (2.94)	2.56 (0.97)
Neutrophil count decreased	8	11.21 (5.59–22.48)	11.14 (73.9)	11.14 (5.56)	3.48 (1.42)
Pleural effusion	7	6.18 (2.94–12.98)	6.14 (30.18)	6.14 (2.92)	2.62 (0.88)
Skin exfoliation	7	4.42 (2.1–9.28)	4.39 (18.38)	4.39 (2.09)	2.14 (0.6)
Cytomegalovirus infection	7	22.79 (10.84–47.91)	22.65 (144.88)	22.65 (10.77)	4.5 (1.59)
Hypoxia	6	9.53 (4.27–21.26)	9.48 (45.56)	9.48 (4.25)	3.25 (1.01)
Septic shock	6	7.6 (3.41–16.95)	7.56 (34.19)	7.56 (3.39)	2.92 (0.87)
Hypogammaglobulinaemia	5	46.98 (19.51–113.13)	46.79 (223.91)	46.76 (19.42)	5.55 (1.26)
Cytomegalovirus infection reactivation	5	84.98 (35.29–204.68)	84.62 (412.7)	84.52 (35.09)	6.4 (1.32)
Haemophagocytic lymphohistiocytosis	5	28.24 (11.73–68)	28.12 (130.77)	28.11 (11.68)	4.81 (1.17)
Neurotoxicity	4	12.89 (4.83–34.4)	12.85 (43.7)	12.84 (4.81)	3.68 (0.64)
Bacteraemia	4	18.89 (7.08–50.42)	18.83 (67.51)	18.82 (7.05)	4.23 (0.75)
Tumour lysis syndrome	4	25.49 (9.55–68.04)	25.4 (93.75)	25.39 (9.51)	4.67 (0.82)
Immune effector cell-associated neurotoxicity syndrome	4	55.11 (20.64–147.13)	54.92 (211.6)	54.88 (20.55)	5.78 (0.93)
Liver function test increased	4	9.68 (3.63–25.84)	9.65 (31.03)	9.65 (3.62)	3.27 (0.53)
Uveitis	3	12.67 (4.08–39.36)	12.64 (32.17)	12.64 (4.07)	3.66 (0.25)
Rash maculo-papular	3	7.52 (2.42–23.34)	7.5 (16.9)	7.5 (2.41)	2.91 (0.07)
Hypophosphataemia	3	22.62 (7.28–70.27)	22.57 (61.83)	22.56 (7.26)	4.5 (0.37)

95% CI, 95% confidence interval; χ^2^, chi-squared; IC, information component; IC025, the lower limit of 95% CI of the IC; EBGM, empirical Bayesian geometric mean; EBGM05, the lower limit of 95% CI of EBGM.

#### 3.3.1 Confirmed known safety signals

The most frequently reported signals were consistent with the established safety profile of mosunetuzumab. CRS was the most common adverse event, with 118 reports. Respiratory failure, hypoxia, pyrexia and chills were also frequently reported. Additional well-characterized immune-related and on-target events consistent with the known safety profile of T-cell engaging bispecific antibodies were also identified, including tumour flare (12 reports), neurotoxicity and immune effector cell-associated neurotoxicity syndrome (ICANS, 4 reports). Hematologic toxicities included neutropenia, febrile neutropenia, and neutrophil count decreased. Injection site and infusion-related reactions were prominent, with injection site reaction and infusion related reaction meeting signal criteria. Hepatobiliary events also emerged as a notable finding, with signals for alanine aminotransferase increased, aspartate aminotransferase increased, and liver function test increased, suggesting potential hepatotoxicity. Immune system disorder signals were identified, hypogammaglobulinaemia and haemophagocytic lymphohistiocytosis both demonstrated with strong signal strengths. Moreover, two cutaneous adverse event signals were identified, including skin exfoliation and rash maculo-papular. Metabolic and nutritional disorders included hypophosphataemia and tumour lysis syndrome. In addition,a substantial number of infection-related signals were identified, which included COVID-19 pneumonia, COVID-19, pneumonia, pneumonitis, pleural effusion, sepsis, septic shock, cytomegalovirus infection and bacteraemia. Cytomegalovirus infection reactivation was also detected, which highlighting the risk of viral reactivation during therapy.

#### 3.3.2 Potential emerging signals

In addition to the positive signals of the above-mentioned known adverse events, a number of PTs were identified as novel potential safety signals, which have not been clearly characterized in the current FDA-approved prescribing information of mosunetuzumab, which we describe in detail below grouped by SOC category. In the cardiac disorders, atrial fibrillation was the only signal that met the positive criteria of the four disproportionality algorithms; Moreover, uveitis was the only positive signal in the eye disorders which needed close clinical attention.

### 3.4 Time-to-onset analysis of AEs

**FIGURE 4:**
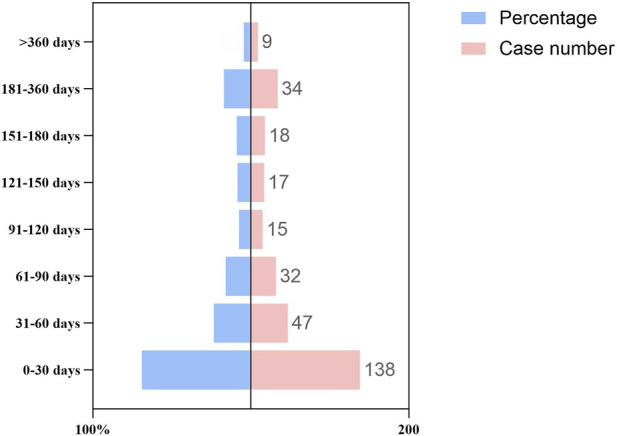
Time-to-onset distribution of mosunetuzumab-associated adverse events in FAERS.

To characterize the temporal occurrence pattern of mosunetuzumab-related adverse events and inform clinical risk monitoring strategies, we performed a predefined time-to-onset (TTO) analysis. TTO was defined as the time interval between the initiation of mosunetuzumab treatment and the onset date of the adverse event. The time-to-onset distribution of AEs was analyzed based on 310 reports with clearly documented event dates (Figure 4). The analysis revealed that most AEs (n = 138) developed within 30 days. Furthermore, 34 cases of delayed AEs were reported after 180 days of therapy.

## 4 Discussion

Immunotherapy represents a significant development in the management of hematological malignancies. Mosunetuzumab, as a bispecific antibody, facilitates targeted killing of FL B cells by engaging and activating T cells (Labanca et al., 2024). The safety profile of mosunetuzumab is comprehensively characterized in this large-scale real-world pharmacovigilance study, utilizing disproportionality analysis of FAERS data. The study identified known risks, including CRS, hematologic toxicities, tumour flare, injection site reaction, infusion related reaction, neurotoxicity, hepatobiliary events, immune system disorders and infection-related events, while also identifying potential new safety signals associated with cardiac and eye disorders, thereby warranting further investigation.

Our results align with previous clinical studies finding that CRS, hematologic toxicities, injection site reaction and infusion related reaction are prominent adverse reactions (Goto et al., 2025; Lewis et al., 2026). CRS remains the most common adverse event associated with mosunetuzumab. The results from the YO43555 trial are fully consistent with our findings (Li et al., 2025). In addition, the GO29781 trial reported pyrexia (100%) and chills (58%) as the most common concomitant symptoms with CRS; grade ≥3 CRS occurred in 2.2% of patients, accompanied by hypoxia and hypotension (Budde et al., 2022). The pathogenesis of CRS has been linked to rapid elevation of inflammatory cytokines following T-cell activation, with interleukin-6 (IL-6), tumor necrosis factor-α (TNF-α), and interferon-γ (IFN-γ) implicated as core mediators (Li et al., 2025; Budde et al., 2022). Neutropenia is one of the adverse reactions to mosunetuzumab (Munakata et al., 2023). Moreover, tumour flare and ICANS were identified as two strong positive signals, which are consistent with the results of real-world (Lewis et al., 2026; Dani et al., 2025). The pathogenesis of these events may involve inflammatory cytokines such as IL-6, though unverified; nonetheless, stepwise dosing and prophylactic medication are recommended to mitigate risk (Géraud et al., 2024). Immune system-associated adverse events include hypogammaglobulinaemia and haemophagocytic lymphohistiocytosis (HLH). The former is a common adverse reaction, while only isolated case reports of the latter have been documented to date. The development of hypogammaglobulinaemia is widely recognized as a class effect of CD20-targeted therapies, which is strongly correlated with an increased risk of infectious complications (Jóna et al., 2026; Alvarez et al., 2023). HLH is an extremely rare adverse event; real-world clinical data have indicated that it is frequently accompanied by excessive T-cell activation and cytokine storm (Holloway et al., 2024). however, no definitive biological evidence to validate this mechanistic association. Among skin and subcutaneous tissue disorders, we found two positive signals: skin exfoliation and rash maculo-papular. They have not been systematically described in the phase 2 registered trial (Budde et al., 2022). Cutaneous adverse events may reflect T-cell activation and inflammatory cytokine release, though mechanistic studies are warranted (Brönnimann and Yawalkar, 2005); nonetheless, vigilant monitoring and timely intervention are advised. Furthermore, we identified hypophosphatemia as an adverse reaction signal, with 17 cases reported in the pivotal GO29781 phase 2 trial (Budde et al., 2022). Serum phosphate fluctuations, which coincide with CRS manifestations, have been proposed as a potential predictive biomarker, though this association awaits confirmation in dedicated studies (Nakamura et al., 2023).

We identified a range of infection-related events as high-frequency positive signals, which covered pneumonia, pleural effusion, septic shock, sepsis, and cytomegalovirus infection. Our findings align closely with the incidence and spectrum reported in previous studies (Sun and Romancik, 2025). Furthermore, two published reports have highlighted the broad clinical burden of infections: one reported a fatal event due to human herpesvirus-6 (HHV-6) infection, the other described chronic persistent parvovirus B19 infection (Samperio et al., 2025; Füreder et al., 2025). Additionally, a fatal case of pleural effusion has been reported with Epcoritamab, an alternative CD20×CD3 bispecific antibody. This event was linked to epcoritamab’s on-target pleural activity, potentially contributing to local CRS (Takahata et al., 2025), but remains mechanistically unvalidated. Expert guidelines recommend withholding mosunetuzumab during active infection until resolution, with routine immunoglobulin monitoring in uninfected patients (Crombie et al., 2024).

Beyond established adverse events, we identified potential safety signals across several SOCs, including cardiac and eye disorders. For cardiac events, atrial fibrillation was the only positive signal. Despite its low reported incidence in real-world cohorts, this signal warrants ongoing clinical monitoring (Sayed et al., 2024). The onset of atrial fibrillation may be mediated by CRS-driven systemic inflammation or indirect immune pathways, though direct biological evidence remains lacking (Sayed et al., 2024; Munir et al., 2024). Uveitis was the sole ocular signal detected. Importantly, FL itself can involve the ocular adnexa, most commonly the conjunctiva, lacrimal gland, and orbit. However, direct lymphomatous infiltration of the uveal tract is extremely rare (Rasmussen et al., 2014; da Cunha et al., 2014). In patients developing uveitis during mosunetuzumab therapy, both drug-induced inflammation and ocular adnexal lymphoma involvement should be considered in the differential diagnosis.

The TTO analysis reveals a distinct biphasic pattern of AE distribution, with most AEs occurring within 30 days, whereas a proportion emerges following 180 days of therapy. This temporal profile highlights the significance of intensive early monitoring and continued vigilance throughout the treatment course. Based on these findings, we suggest a stratified monitoring approach. During initial treatment and dose escalation, enhanced surveillance is advised, encompassing vital signs, complete blood count with differential, inflammatory markers (e.g.,,C-reactive protein, interleukin-6), immunoglobulin levels, and screening for viral reactivation (e.g., hepatitis B, cytomegalovirus). Patients with recurrent infections, prior transplantation, or hypogammaglobulinaemia risk factors may warrant individualized, more frequent assessment. In cases of febrile neutropenia, severe cytokine release syndrome, catheter-related bloodstream infection, or opportunistic infections, multidisciplinary consultation with infectious disease, pharmacy, and critical care specialists should be considered. Finally, based on immune status, timely prophylactic measures—including acyclovir, trimethoprim-sulfamethoxazole, and vaccination against influenza and pneumococcus—are warranted (Longhitano et al., 2021). This stratified monitoring approach facilitates early recognition of immune-related adverse events and potential late-onset infectious complications, thereby optimizing the risk-benefit profile of mosunetuzumab in real-world clinical practice.

To assess whether the inclusion of pre-approval clinical trial data (reports before 2023) influenced our primary findings, we conducted a sensitivity analysis comparing adverse event reporting proportions between the period after 2023 (post-approval real-world reports) and the full study period (2004–2025, including trial data). As shown in Supplementary Table S1, the reporting proportions of key adverse events—including cytokine release syndrome, hematologic toxicities, infection-related events, and hepatobiliary events—did not differ significantly between the two periods (all P > 0.05). These results support the robustness of our primary disproportionality analyses.

Several limitations inherent to the pharmacovigilance methodology should be considered when interpreting our findings. First, rather than establishing definitive causal relationship, disproportionality analysis can only determine statistical associations between drug exposure and AEs. Second, the FAERS, as a spontaneous reporting system, is subject to inherent limitations, including underreporting, reporting biases, and potential incompleteness of case information. Third, confounding by concomitant medications and underlying disease progression could not be fully adjusted. Moreover, the absence of precise event onset dates in a subset of reports may impact the robustness of time-to-onset analyses and consequently the accurate interpretation of certain safety signals.

In conclusion, data mining of the FAERS database demonstrates that the real-world safety profile of mosunetuzumab encompasses established risks (CRS, neutropenia and injection site reactions) and potential safety signals involving cardiac and eye systems. Although most AEs occur during early treatment, a proportion emerges during long-term therapy, necessitating sustained clinical vigilance. These findings support stratified risk assessment and phase-appropriate monitoring implementation in clinical practice. Considering the inherent limitations of spontaneous reporting systems and disproportionality analysis methodologies, the signals identified in this study warrant further validation and mechanistic investigation through prospective cohort studies, registry data, and dedicated translational research.

## Data Availability

The original contributions presented in the study are included in the article/Supplementary Material, further inquiries can be directed to the corresponding authors.
