# Supplementary material for: Real-world safety profile of mosunetuzumab: a pharmacovigilance study based on the food and drug administration adverse event reporting system
Source: Front Pharmacol. 2026 May 26;17:1809879. doi: 10.3389/fphar.2026.1809879 (PMC13246391; doi:10.3389/fphar.2026.1809879)
Supplement: Supplementary file 1 [file Table1.docx]

Supplementary Table S1. Comparison of adverse events reporting proportions before and after 2023

| Adverse event | After 2023 (N=1029) | 2004-2025  （N=1154） | P value* |
| --- | --- | --- | --- |
| Cytokine release syndrome | 109 | 118 | 0.779 |
| Hematologic toxicities | 30 | 42 | 0.344 |
| Injection site reaction and Infusion related reaction | 22 | 22 | - |
| Nervous system disorders | 4 | 8 | 0.337 |
| Infection related events | 120 | 143 | 0.601 |
| Hepatobiliary events | 27 | 27 | - |
| Immune system disorder | 10 | 10 | - |

*P value was calculated using the chi‑square test. “–” indicates that the test was not performed because the counts were identical between the two periods.

Note: Adverse event categories were defined based on the Preferred Terms (PTs) that met the positive signal criteria in the primary disproportionality analysis (see Table 5). The composition of each category is as follows: Cytokine release syndrome: cytokine release syndrome.Hematologic toxicities:neutropenia, febrile neutropenia, neutrophil count decreased.Injection site reaction and Infusion related reaction:injection site reaction, infusion related reaction.Nervous system disorders:neurotoxicity,immune effector cell‑associated neurotoxicity syndrome (ICANS).Infection related events: COVID‑19 pneumonia, COVID‑19,pneumonia, pneumonitis, pleural effusion, sepsis, septic shock,cytomegalovirus infection,bacteraemia,cytomegalovirus infection reactivation.Hepatobiliary events:alanine aminotransferase increased,aspartate aminotransferase increased, liver function test increased.Immune system disorder: hypogammaglobulinaemia, haemophagocytic lymphohistiocytosis.
